# Natural and experimental evolution of sexual conflict within *Caenorhabditis* nematodes

**DOI:** 10.1186/s12862-015-0377-2

**Published:** 2015-05-22

**Authors:** Michael F. Palopoli, Colin Peden, Caitlin Woo, Ken Akiha, Megan Ary, Lori Cruze, Jennifer L. Anderson, Patrick C. Phillips

**Affiliations:** Department of Biology, Bowdoin College, ME 04011 Brunswick, USA; Institute of Ecology and Evolution, University of Oregon, OR 97403 Eugene, USA; Current address: South Lane School District, OR 97424 Cottage Grove, USA; Current address: Department of Obstetrics and Gynecology, Medical University of South Carolina, SC 29412 Charleston, USA; Current address: INRA, UR1037 LPGP, Campus de Beaulieu, F-35000 Rennes, France

**Keywords:** Experimental evolution, Mating systems, Sexual conflict, Sexual selection, Sperm competition

## Abstract

**Background:**

Although males and females need one another in order to reproduce, they often have different reproductive interests, which can lead to conflict between the sexes. The intensity and frequency of male-male competition for fertilization opportunities is thought to be an important contributor to this conflict. The nematode genus *Caenorhabditis* provides an opportunity to test this hypothesis because the frequency of males varies widely among species with different mating systems.

**Results:**

We find evidence that there is strong inter- and intra-sexual conflict within *C. remanei*, a dioecious species composed of equal frequencies of males and females. In particular, some *C. remanei* males greatly reduce female lifespan following mating, and their sperm have a strong competitive advantage over the sperm of other males. In contrast, our results suggest that both types of conflict have been greatly reduced within *C. elegans*, which is an androdioecious species that is composed of self-fertilizing hermaphrodites and rare males. Using experimental evolution in mutant *C. elegans* populations in which sperm production is blocked in hermaphrodites (effectively converting them to females), we find that the consequences of sexual conflict observed within *C. remanei* evolve rapidly within *C. elegans* populations experiencing high levels of male-male competition.

**Conclusions:**

Together, these complementary data sets support the hypothesis that the intensity of intersexual conflict varies with the intensity of competition among males, and that male-induced collateral damage to mates can evolve very rapidly within populations.

## Background

Males and females typically have different reproductive strategies [1, 2], which often result in conflicts surrounding control of fertilization [3–5]. Because females invest more in each offspring, males usually produce an excess of gametes and are often driven to compete with each other and to manipulate their potential mates in order to fertilize as many eggs as possible. In some cases, males are expected to evolve strategies that actually harm their female partner, as long as the associated increase in male fertilization success is sufficiently high. Females, meanwhile, evolve to avoid manipulation by males by optimizing the time, place, and partner for each fertilization. The resulting antagonistic coevolution of strategies and counter-strategies can be characterized as a perpetual “three-way tug-of-war” over control of fertilization-male versus male versus female-in which sustained antagonistic coevolution drives rapid change [6].

Conflicts over control of fertilization are thought to explain the evolution of a wide variety of morphological, behavioral, and molecular traits related to fertilization success in both males and females (reviewed in [4]). For example, antagonistic coevolution is thought to explain the observation that male fruit flies transfer a complex suite of accessory-gland proteins to females during insemination, some of which can alter the reproductive patterns of their mates in ways that reduce female fitness while enhancing sperm competitive ability (reviewed in [7]). By generating flies lacking these proteins, it is possible to demonstrate benefits to males producing them (*e.g.*, increased probability of paternity), and costs to females receiving them (*e.g.*, shifts in reproduction and/or reduced female life span; [1, 2, 8]). Comparisons of accessory gland proteins within and between fruit fly species have suggested that they often evolve rapidly due to natural selection [9], which is consistent with the hypothesis that these proteins tend to evolve due to sustained, antagonistic coevolution.

Conflicts over control of fertilization have also been revealed by manipulating the mating system of a species in the laboratory and then observing the resulting evolution of interactions within and between the sexes (reviewed in [10]). For example, when female fruit flies are prevented from coevolving with their mates, the males adapt rapidly to the static female phenotype, becoming more successful competitors when mating with those particular females, and also causing increased collateral damage to female survivorship [11]. On the other hand, when conflicts over control of fertilization are eliminated entirely-by choosing random, monogamous pairings each generation-male fruit flies evolve to be less harmful to their mates, and females evolve to be less resistant to male-induced harm [12]. These results suggest that the promiscuous mating system normally found in this species has created a conflict load that can be relieved quickly in response to enforced monogamy.

While these insights from fruit flies have been invaluable, they exist within a background of a fixed male–female mating system. In contrast, nematodes in the genus *Caenorhabditis* provide a particularly unique opportunity to test models of sexual conflict, since the frequency of males varies widely among species with different mating systems. For example, conspecific matings between hermaphrodites from the self-fertilizing (low male) *C. briggsae* and males from the outcrossing (high male) *C. nigoni* can lead to *C. nigoni* sperm breaking free of the *C. briggsae* spermatheca and wandering around in the hermaphrodites body—to ill effect [13]. Similarly, populations of the outcrossing *C. remanei* consist of males and females at roughly equal frequencies, and mating is promiscuous, at least in the laboratory. Furthermore, patterns of genetic variation within and among *C. remanei* populations are consistent with dioecious mating and regular outcrossing [14–16]. As a result, we can predict that male-male competition for fertilization opportunities will be intense, and there are likely to be strong male–female conflicts over control of fertilization. Documented phenotypes that are consistent with a high level of male-male competition in *C. remanei* include: males produce large amoebic sperm, which presumably makes those sperm more competitive [3–5, 17]; males deposit robust copulatory plugs, which interfere with subsequent mating attempts by rival males ([18], see also [19]); females produce a highly effective sex pheromone that attracts males, creating greater opportunity for male-male competition [20]; and females exhibit mating torpor—remaining still during male mating attempts—which presumably makes it feasible for nearby males to compete directly for mating opportunities [21]. Furthermore, Diaz et al. [22] demonstrated that females suffer reduced survival when faced with large numbers of males, which is consistent with the existence of a conflict load in this species.

In contrast, *C. elegans* populations appear to consist almost entirely of self-fertilizing hermaphrodites, with only the occasional male available for outcrossing. Extremely low male frequencies are typically observed in the laboratory (*e.g.*, [23]), and male frequencies appear to be even lower in field populations [24]. Furthermore, *C. elegans* does not seem to suffer from inbreeding depression, which is consistent with the hypothesis that repeated self-fertilization has rid the gene pool of most deleterious, recessive alleles [25]. Finally, patterns of genetic variation in this species suggest that outcrossing with males rarely occurs within natural populations, perhaps just once in every thousand generations [14–16, 24, 26, 27].

Phylogenetic and genetic evidence together suggest that the *C. elegans* mating system is likely to have evolved from a dioecious ancestor within the last few million years [26]. During that time period, it appears that the decrease in mating frequency has altered the expression of a suite of sexual interaction traits. For example, the males from many natural isolates of *C. elegans* have lost their ability to produce copulatory plugs, and this variation is due to a loss-of-function mutation that appears to have spread worldwide recently [18]. In addition, hermaphrodites within *C. elegans* appear to minimize interactions with males by not producing any high-potency, attractive mating pheromones [20, 28]. Finally, not only do *C. elegans* hermaphrodites fail to display the mating-torpor response seen in their closely related dioecious relative *C. remanei*, but they often move away rapidly from males who are attempting to mate [21]. Overall, it appears that *C. elegans* has evolved a suite of changes in mating traits that are consistent with a low degree of outcrossing, which suggests that levels of male-male competition have been extremely low in the recent past.

Since the strength of sexual conflict is expected to vary with the frequency of male-male competition, we can predict that the arms race surrounding control of fertilization should vary dramatically among species in the genus *Caenorhabditis* (e.g. [13]). Species with the ancestral dioecious mating system, such as *C. remanei*, should exhibit signs of an ongoing, intense conflict over control of fertilization opportunities; in contrast, species that have evolved to become primarily self-fertilizers, such as *C. elegans*, should have evolved towards a very different mating dynamic, in which male-male competition is almost entirely absent, and so male-induced harm of mates should have evolved to become greatly reduced. Here we test these predictions in two ways. First, we examine both inter- and intra-sexual interactions within *C. remanei* and demonstrate the existence of sexual conflict within this species. Second, we use mutations that transform the mating system within *C. elegans* back to a dioecious system and demonstrate that the same hallmarks of sexual conflict as are observed within *C. remanei* can be recapitulated over a period of just a few dozens of generations of evolution in the presence of elevated male sexual encounters. Together, these results demonstrate that evolution of sexual conflict is dependent upon mating frequency and that this group of nematodes provides a powerful system for addressing hypotheses related to the evolution of sexual interactions.

## Results

### Mating and reproduction in *C. remanei*

**Fig. 1:**
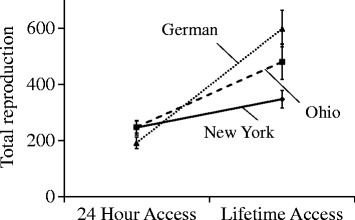
Male–female effects on reproductive output for three natural isolates of *Caenorhabditis remanei* (New York = EM464, Ohio = PB279, German = SB146). Average reproduction for females mated with males for 24 h or for their full lifetimes. Increases in reproductive output indicate that females are sperm limited, but that the increases are generated in an isolate-specific manner

In order to assess the opportunity for male-male and male–female interactions within the obligately-outcrossing species, *C. remanei*, we examined variation in offspring production and reproductive success across three natural isolates: EM464, originally isolated from New York State, PB259 from Ohio, and SB146 from Germany [29]. For clarity we use these geographic names in addition to the strain names when reporting the results. As indicated by the increase in total offspring production with prolonged access to mates, females of *C. remanei* are sperm-limited, requiring multiple matings to fertilize all of their eggs (Fig. 1). Averaging over all of the crosses, females with 24-h access to males had mean lifetime fecundity (n = 122, mean = 246 ± 11 offspring) that was two-fold less than females that received lifetime access to males (n = 144, mean = 483 ± 18 offspring; *F*_1,228_ = 222.30, *p* < 0.0001). Males differed in their impact on female reproductive success, but all males had increased reproductive output with prolonged access to females (*F*_*2, 265*_ = 13.57, *p* < 0.0001; Fig. 1). With 24-h access to females, New York (EM464) males and Ohio (PB259) males had more offspring, on average, than German (SB146) males, but did not vary significantly from each other in mean reproductive output (Tukey HSD). With lifetime access to females, there was a dramatic interaction between male strain and reproductive success (Fig. 1; *F*_2, 248_ = 30.97, *p* < 0.0001)—German males generated significantly more offspring than Ohio males, which in turn performed better than New York males (Tukey HSD).

### Sperm competition in *C. remanei*

**Fig. 2:**
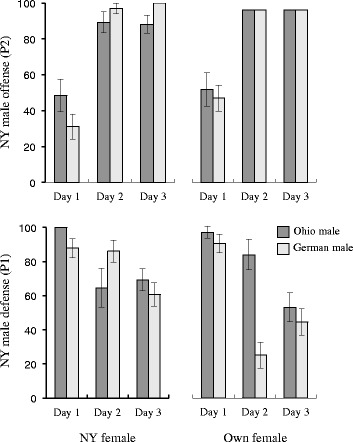
Sperm competitive ability of New York (EM464) males relative to males from Ohio (PB279) and Germany (SB146). Each graph shows the percentage of offspring fathered by New York males relative to a single competitor. Top panels show the New York male “offense” (P2) after being introduced to a female for 24 h following her being mated with another male for the previous 24 h (i.e., there are no males on the plate on days 2–3). Bottom panels show the opposite or “defense” (P1) response where the New York males were introduced first and then replaced by males from either Ohio or Germany. Panels on the left show results when females from the New York isolate were the mating partners and panels on the right show results when the female matched the non-New York male competitor. Error bars represent standard errors of the binomial frequency estimates

Although some males are more successful than others in terms of total offspring production when solely mated with a female, it is possible that reproductive success in the absence of competition trades off against competitive ability when other males are present. Indeed, when directly competed against other males, sperm from New York (EM464) males significantly outperformed the sperm from males from Ohio (PB259) and Germany (SB146) (Fig. 2; Fisher’s Exact Test *p* < 0.001 for all comparisons except NY-German Day 2, *p =* 0.0430). When mated second, sperm from New York males rapidly displaced sperm from the other two males, siring nearly 100 % of offspring after the first day of competition (Fig. 2). This effect was not dependent upon the genetic background of the female. In contrast, sperm from Ohio and German males was relatively ineffective at displacing New York male sperm when the New York males mated first, with New York males accounting for 45-70 % of offspring produced even three days after they were last in contact with the female. The only indication of a male-by-female interaction was the slightly higher success on day 2 of German males when mating as the second male with a female from their own population. This effect was not consistent across the entire time-course, however (Fig. 2).

**Fig. 3:**
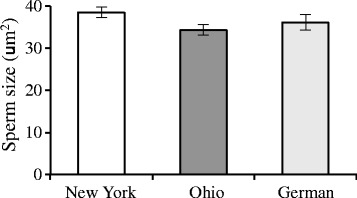
Average sperm size for the males from three natural isolates of *C. remanei*. The average difference among strains is not significant, as most of the variation in sperm size is do to variation within (81 %) or between (18 %) males within strains

These strong differences in competitive ability could not be readily explained by differences in sperm size among the males. Although EM464 had slightly larger sperm than males from the other strains, these differences were not significant (Fig. 3; *F*_2,24_ = 1.40, *p* = 0.2636). Overall, differences among the strains only accounted for less than 1 % of the total variation in sperm size, while variation among males within strains explained 18 % of the variation (which is significant; *F*_24,1615_ = 13.71, *p* < 0.0001).

### Mating effects on longevity in *C. remanei*

**Fig. 4:**
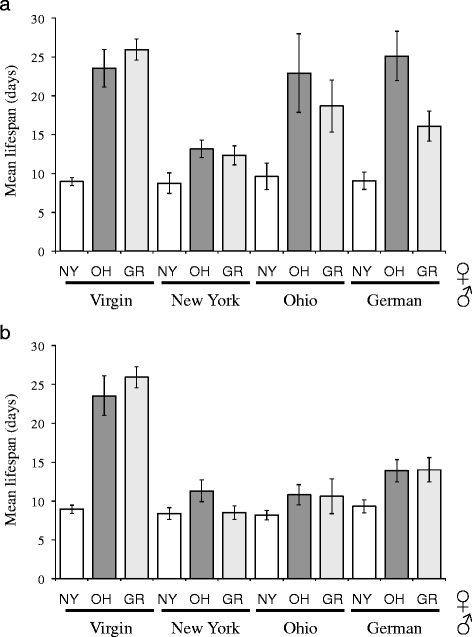
Effects of males on average female lifespan for different combinations of isolates of *C. remanei*. Females from New York tend to have short lifespans under all conditions, while the lifespan of the other females is strongly dependent on which male they mate with. (**a**) Female lifespans following 24 h of mating with males on the first day of adulthood. (**b**) Female lifespans when mated with males over the entire course of their lives

Models of the coevolution of sexual antagonism between males and females suggest that variation in male reproductive success can tradeoff against female reproduction, as seen above, as well as against female survivorship. For these crosses, female longevity depended strongly on strain specific interactions between males and females (Fig. 4; *F*_4, 248_ = 6.73, *p* < 0.0001). New York (EM464) females displayed a short lifespan of roughly nine days, whether mated or unmated. In contrast, mating reduced overall lifespan for both Ohio (PB259) and German (SB146) females in a male-specific manner (*F*_2, 248_ = 132.82, *p* < 0.0001). In particular, females exposed to New York males for even 24 h lived half as long as they did when unmated (Fig. 4a). Mating with Ohio and German males was also detrimental, although lifespan was only reduced by 20 % in these cases (Fig. 4a). Mating with males for their lifetime further reduced female lifespan for all isolates, but for the most part, the bulk of a male’s effect (50-90 %) on female lifespan was achieved within the first 24 h (Fig. 4b).

### Experimental evolution of sperm competition in *C. elegans*

*C. elegans* normally reproduces as self-fertilizing hermaphrodites, with occasional outcrossing via rare males [23]. We used the *fog-2* mutation [30], which blocks sperm production in hermaphrodites and effectively feminizes them, to convert the androdieocious mating system of *C. elegans* to resemble that of *C. remanei* in a single step [31]. This mutation was backcrossed and made homozygous into 12 different *C. elegans* natural isolates, which were crossed together for six generations and then maintained in three replicates for 60 generations of experimental evolution as male “high-competition” lines. The same set of isolates, this time lacking the *fog-2* mutation, where also maintained for 60 generations as male “no-competition” lines (no males were maintained in those populations during the course of the experiment).

**Fig. 5:**
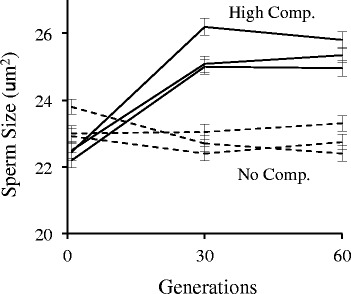
Effects of 60 generations of experimental evolution in *C. elegans* under high-competition (outcrossing) and no-competition (self-reproduction) conditions on sperm size. Increased male-male completion leads to a rapid increase in male sperm size, although this effect rapidly asymptotes and the size of these sperm is still much smaller than that observed in *C. remanei*. Error bars represent on SEM

High-competition males evolved significantly larger sperm than no-competition males (contrast between G0 and G60 lines, *F*_1,8_ = 37.80, *p* = 0.0003, Fig. 5). The majority of this response occurred within 30 generations, however, with little change observed between the G30 and G60 lines (*F*_1,8_ = 0.01, *p* = 0.9654). Indeed, the G30 and G60 lines were more similar than one would expect by chance alone, which is consistent with an expectation of fixation of standing genetic variation within these lines. There was no evidence for significant heterogeneity in response among the replicate lines within each treatment (*F*_4,8_ = 1.63, *p* = 0.2568). Even with a 10-15 % increase in size under experimental evolution, the size of *C. elegans* sperm remained 40 % smaller than *C. remanei* sperm, even though these two species have very similar body sizes.

**Fig. 6:**
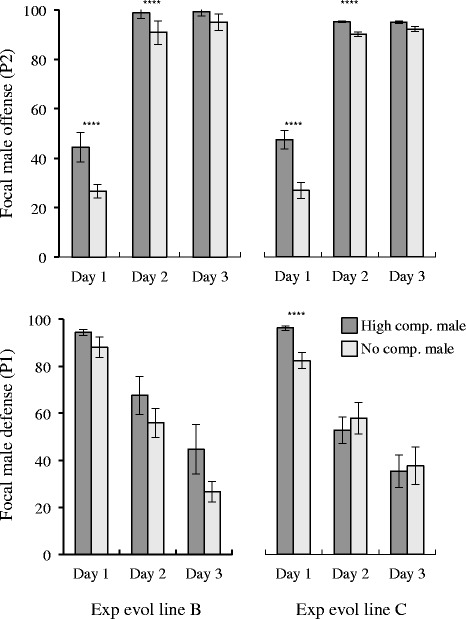
Sperm competitive ability for two lines of high- and no-competition *C. elegans* experimental evolution lines. Format is the same as Figure 2, except that here males from each experimental treatment are compared to a standard GFP-marked male and significance testing is therefore based on performance differences between experimental evolution treatments. Error bars represent approximate standard errors based on among-plate estimates of mating success. ****p < 0.0001

Consistent with the changes in sperm size, high-competition males were able to outcompete the sperm deposited by the GFP-tester males significantly better than no-competition males (Fig. 6). We tested sperm competition in lines that both showed and did not show negative effects on female longevity (below, lines B and C, respectively). Interestingly, in addition to evolving larger size, sperm from these lines also evolved to be more competitive in “offense” relative to the no-competition controls when introduced as the second male (logistic regression *p* < 0.0001 for days 2 and 3, after accounting for among replicate error; Fig. 6). Sperm from the high competition lines also tended to do better than the no-competition sperm in defense on average, although the result was only statistically significant for Line C during the first day of competition (*p <* 0.0001; Fig. 6).

### Experimental evolution of male–female interactions in *C. elegans*

**Fig. 7:**
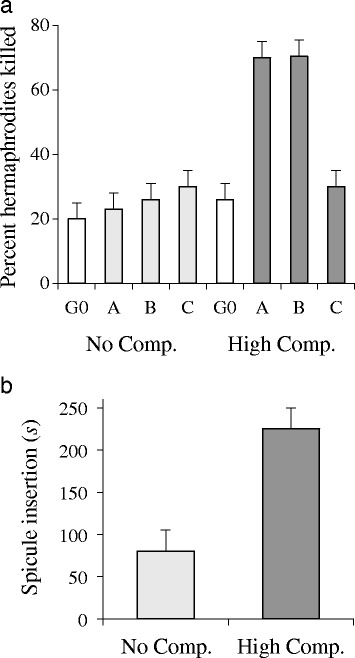
Evolution of male–female interactions under 60 generations of experimental evolution in *C. elegans* under high and no male-competition conditions. (**a**) Percentage of tester hermaphrodites who failed to survive a five-day period with focal males. Two of three high-competition lines showed significantly increased mortality. (**b**) Time spent during spicule insertion during successful matings for the high- and no-competition males. Error bars are one SEM

*C. elegans* hermaphrodites were twice as likely to die in mating trials with males from high male competition lines than they were when mating with those males’ pre-selection ancestors (Fig. 7a; *F*_1,41_ = 11.41, *p* < 0.0001). This effect evolved in two of the three replicate lines (Dunnett’s Method, control vs: A & B, *p* = 0.0004; C, *p* = 0.8889). In contrast, hermaphrodite mortality did not significantly increase upon mating with the no-competition control lines (Fig. 7a; *F*_1,38_ = 2.55, *p* = 0.0697). These mortality effects were correlated with changes in mating behavior, specifically the total time of spicule insertion, which was nearly four times longer in the high competition lines than it was in the no-competition lines (Fig. 7b; *t*_13_ = 4.24, *p* = 0.0009; Wilcoxon *p* = 0.0016).

## Discussion

The opportunity for selection on sexual interaction traits should vary with the frequency of these interactions. In particular, a tendency for females to mate with multiple males has the potential to increase the variance in mating success among males, and provides the opportunity for strong pre- and post-copulatory competition among males [4]. Within the nematode genus *Caenorhabditis*, the opportunities for male-male competition seem likely to vary substantially among species. For example, Ting et al. [13] found that hermaphrodities in the self-fertilizing species *C. briggsae* are poorly resistant to the harmful effects of mating with males from the closely related male–female species *C. nigoni.* The dioecious species *C. remanei* consists of males and females in roughly equal frequencies, and exhibits patterns of genetic variation consistent with frequent outcrossing (e.g., [16, 32]), so it seems likely that males are competing regularly for mating opportunities. In contrast, its androdioecious relative *C. elegans* appears to reproduce almost entirely by self-fertilization, with males typically existing at such low frequencies that it seems unlikely that males will end up competing directly very often for mating opportunities [23,24]. Here we investigate mating competition in both of these species and find results consistent with theoretical expectations: (1) *C. remanei* displays strain-specific sexual interaction traits that are indicative of an evolutionary history of inter- and intrasexual conflict; and (2) by shifting the mating system of *C. elegans* to resemble that of *C. remanei*, we are able to recapitulate these attributes in just 60 generations of experimental evolution.

The existence of sperm-limitation and multiple mating within *C. remanei* (Fig. 1; see also [22]) suggest the opportunity for strong sexual selection among males and thereby the potential for sexual conflict [33]. The existence of sperm competition can itself lead to the evolution of sperm-limitation, as selection on characteristics that enhance sperm competitive ability are balanced against those that enhance total fertilization in the absence of competition [34]. In keeping with this, the clearest signal that we observe for male-male competition was revealed in strong strain-specific differences in sperm competitive ability, with males from the New York (EM464) strain displaying clear competitive superiority to that from the Ohio (PB259) and German (SB146) strains in both offensive and defensive ability (Fig. 2).

The amoeboid sperm of *Caenorhabditis* nematodes is very large relative to the body size of the nematodes [17] and is actually several times the size of most mammalian sperm. Larger sperm are known to crawl faster and outcompete smaller sperm consistently, perhaps due to direct physical interactions within the spermatheca in which sperm are stored [35]. Although sperm from New York males is slightly larger that of the other males, they are not distinctly different (Fig. 3), and there is a great deal of overlap in the size distributions. We do not currently know enough about what makes an individual sperm more competitive to know if these slight size differences are sufficient to explain the differences in competitive ability that we observe here.

The existence of strain-specific effects on female reproduction and longevity that we observe here are strongly indicative that the opportunity for sexual conflict reflected in male-male competition has led to the evolution of male–female conflict within this species. Because of the likely co-evolution of male and female conflict within populations, crosses between populations can be an especially powerful means of detecting residual sexual conflict [36], which is what we observe here. It is well known that mating can lead to large decreases in longevity within nematodes (e.g., [37]). We have shown that these decreases can occur after relatively brief encounters with males (<24 hr) and in a strain-specific manner. Recent work has demonstrated that merely sensing the presence of males is sufficient to cause a decrease female longevity through the detection of male-specific chemical factors [38] and that seminal fluid can have effects that are independent of sperm [39]. We currently cannot determine if similar chemical factors are at play in our case or if the differences we observe might be caused by direct behavioral interactions between males and females.

These results raise the question of whether there is any correlation between male competitive ability and the associated collateral damage to females during and after matings in *C. remanei*. Interestingly, we found that in *C. remanei* the most competitive males cause the greatest collateral damage to females. In particular, New York males (EM464) were superior to the other two strains when forced to compete directly via sperm competition (Fig. 2), and females mated to New York males died much sooner on average than females mated with males from the other two strains (Fig. 4). This correlation is consistent with the hypothesis that some of the traits conferring an advantage during sperm competition are also causing collateral damage to females. New York females seem immune to the collateral damage of New York males, which is also consistent with the hypothesis of an ongoing arms race between the sexes, although this interpretation is complicated by the fact that New York females tend to have short life spans in general, whether exposed to males or not. Somewhat surprisingly, most of the male-induced damage appears to accumulate during the first 24 h of exposure to males as, while continued exposure to males does further decrease female lifespan, it does not decrease as dramatically as might otherwise be expected (Fig. 4). Fritzsche et al. [40] have recently shown that female *C. remanei* respond more quickly to relaxed sexual selection than males, indicating that females are likely to be under direct pressure from sexual conflict among males. Overall, then, while we currently cannot form a causal link between competition and conflict, the existence of strong male-male and male–female interactions matches our expectations of how these phenomena should emerge based on the structure of the mating system within this species.

Unlike *C. remanei*, everything we know about the androdioecious species *C. elegans* suggests that competition between different males is rare. Indeed, males themselves are extremely rare in *C. elegans*, such that the vast majority of progeny in nature appear to be produced via self-fertilization (e.g., [23, 24]). Furthermore, about half of the strains of *C. elegans* collected from nature worldwide are fixed for a loss-of-function mutation at the *plg-1* gene, which results in males being unable to deposit copulatory plugs after mating [18]. Since the plugs seem to be a mate-guarding adaptation that enhances sperm defense after mating, the worldwide distribution of this mutation suggests that mate-guarding is no longer as useful in *C. elegans* populations as it probably was in the past. Males produce sperm that are barely larger than hermaphroditic sperm, consistent with the hypothesis that males have become specialized in out-competing hermaphrodite sperm but are no longer pushed to produce sperm of larger sizes by intense male-male competition [17]. Finally, *C. elegans* hermaphrodites lack both the mating-torpor response and the potent sex pheromone that are observed in *C. remanei* females [20, 21]. The emerging picture is of a species that has lost many of the traits associated with male-male competition and seems likely to lack the collateral damage to mates that is usually associated with that competition*.*

Interestingly, it appears that many of these traits associated with intense male-male competition and sexual conflict that we observe within *C. remanei* can be recapitulated in *C. elegans* after just 60 generations of evolution under increased male-male competition. For example, we see rapid increases in sperm size in the *fog-*2 obligate outcrossing populations relative to the no-competition controls (Fig. 5). This observation is consistent with an earlier study by LaMunyon and Ward [41], who used different mutations and different lines to establish a very similar experimental framework. They also observed increased sperm size under increased male-male competition. Further, in our populations, males evolved under high-competition conditions demonstrated a significant increase in sperm offense ability (Fig. 6). In other words, the high-competition males were able to outcompete sperm deposited by rival males in a previous mating more effectively than no-competition males. This is a direct test of the hypothesis that *C. elegans* males should evolve greater sperm competitive ability under conditions of enhanced male-male competition. There was weaker evidence for the evolution of increased sperm “defense” (Fig. 6), which may have been caused by the fact that the frequency of transfers in our system did not really require males to protect against rival sperm for a 24 h period, as in our sperm competition assays.

What is the cause of these evolved differences? High-competition males did not mate significantly more frequently with wildtype hermaphrodites, nor did they insert their spicules significantly faster when compared with no-competition males (results not shown). However, in the case of successful matings, high-competition males kept their spicules inserted in the hermaphrodite’s vulva for nearly four times longer than did no-competition males. This evolved copulation period represents roughly 3 % of the worm’s six-day reproductive lifespan per mating, so this is not an insignificant change. Prolonged copulation appears to be a common response to male-male sperm competition, as well as a potentially significant source of sexual conflict [42]. Interestingly, similar to what we observed for *C. remanei*, hermaphrodites died much more frequently over a five day period when exposed to high-competition males than when exposed to no-competition males (Fig. 7). Carvalho et al. [43] observed that, under fairly similar conditions with *fog-*2 mutants, females can eventually evolve increased lifespans (relative to ancestors), indicating that females have the potential to co-evolve in the presence of more harmful males. Overall, these results are consistent with the idea that the competition among males drives negative impacts on females, as we observe for the natural *C. remanei* populations.

The evolved male harm was observed in only two of the three experimental evolution populations, however (Fig. 7). The evolutionary dynamics of sexual conflict are likely to be sensitive to changes in the mating system [5]. Indeed, the outcome may be difficult to predict in any particular example, and seems likely to be sensitive to the genotypes available in the starting population (e.g., [44]). Although the high-competition Line C did not show evidence for evolved male harm, it displayed an increase in sperm size and sperm competitive ability that is comparable to Line B, whose males are significantly more harmful to females than the no-competition controls. This suggests that whatever factors lead to increases in sperm competitive ability can be independent of the evolved harm responses. It is also possible that other mating advantages that are correlated with the female-harm results are not revealed by the assays conducted here. In the case of *C. elegans*, we do not currently have evidence that evolved male responses may or may not have led to changes in female reproductive success. Overall, then, male mating success in both *C. elegans* and *C. remanei* probably relies on a combination of sperm size, male behavior, and other unknown components such as seminal fluid proteins.

## Conclusions

Our results are consistent with previous work on sperm competition and sexual conflict in other taxa, such as *Drosophila melanogaster* [45, 46]. One thing that makes the present work unique is that we were able to demonstrate both ends of the male-male competition spectrum within the same genus and draw a convincing connection between the two states by manipulating the mating system during experimental evolution. Similar “experiments” appear to occur naturally within plant populations in which differential fertilization ability between self-compatible and self-incompatible populations appears to evolve fairly readily, presumably because of increased pollen competition within outcrossing populations [47–49]. Our results demonstrate that the apparent male-hermaphrodite conflict observed in interspecies crosses between *C. briggsae* and *C. nigoni* [13] is also prevalent within species as well and can evolve within a rapid timeframe. In a limited sense, we were able to transform the low-conflict mating system of *C. elegans* into the high-conflict mating system of *C. remanei* after just 60 generations of experimental evolution, highlighting the dynamic nature of these traits. Indeed, the short time frame for the experiment suggests that most of the alleles necessary to transform *C. elegans* towards a system more like *C. remanei* are already segregating within the *C. elegans* gene pool. This is consistent with previous results showing that plugging ability, which is apparently a mate-guarding trait, remains polymorphic within natural populations [18]. It will be interesting to see if other loss-of-function mutations have contributed to the loss of male competitive traits in *C. elegans.* The distinction between these species on this basis provides the opportunity to identify the functional basis of male–female sexual interactions within these groups.

## Methods

### Nematode strains and culture conditions

Nematodes were cultured at 20 °C in Petri dishes containing NGM agar spotted with the *Escherichia coli* strain OP50 using standard protocols [50]. Geographic isolates of both *C. elegans* and *C. remanei* were raised under standardized laboratory generations for >10 generations to minimize maternal or laboratory adaptation effects during experiments. For normal maintenance, stocks were transferred twice weekly to prevent overcrowding and dauer formation. Strains were frozen at −80 °C in a glycerol solution using standard procedures [50].

In *C. remanei*, phenotypes were compared among three geographic isolates: PB259, EM464, and SB146. Both SB146 and EM464 were obtained from the Caenorhabditis Genetics Center (CGC), whereas PB259 was a gift from Scott Baird at Wright State University. These are largely isofemale lines and may have been subject to some inbreeding during derivation, although they are known to harbor substantial residual genetic variation.

In *C. elegans*, twelve geographic isolates served as the initial pool of standing genetic variation for experimental evolution in the laboratory. Strain names, along with the location where originally isolated, were as follows: AB1 and AB2 from Australia; PB303, PB305, PB306, and PB307 from Ohio; CB4855, CB4857, and DH424 from California; CB4856 from Hawaii; CB4932 and N2 from England. Most of these strains were obtained from the CGC, whereas the four “PB” strains were gifts from Scott Baird at Wright State University. Two additional CGC strains were also used: JK574 served as the source of the *fog-2 (q71)* mutation that renders hermaphrodites self-sterile [30]; this mutation was used to prevent self-fertilization in half of the evolving lines (see below). PD4792 served as the source of the transgenic array that expresses high levels of Green Fluorescent Protein (GFP) in the pharynx; this transgene was used to determine paternity in sperm competition experiments involving *C. elegans*.

### Experimental evolution of *C. elegans*

The genetic variation present across the 12 geographical isolates was mixed together to serve as the founding populations for the creation of both High-Competition (HC) and No-Competition (NC) lines for experimental evolution. To create the founding stock for the HC lines, each geographic isolate was first modified to render hermaphrodites self-sterile (*i.e.*, unable to produce their own sperm, thereby needing males in order to reproduce). This was accomplished by introgressing the *fog-2 (q71)* mutation from stock JK574 into each genetic background independently via ten generations of backcrossing. The *fog-2 (q71)* mutation was maintained during these introgressions by selecting for self-sterile hermaphrodites every few generations. Worms from the resulting [*fog-2*] introgression strains were then mixed in equal proportions and maintained in a large population to mate freely for at least six generations, in order to recombine the genomes and thereby create the HC-founding stock. To create the NC-founding stock in parallel, self-fertile hermaphrodites and males from the original twelve geographic isolates were mixed in equal proportions and maintained in a large population to mate freely for at least six generations, in order to recombine the genomes and thereby create the NC founding stock. Both the HC-and NC-founding stocks were frozen for later analysis.

For experimental evolution of HC and NC lines, we essentially replicated the protocol used by [41]. For each of three independent HC lines, 60 L4 self-sterile hermaphrodites and 100 L4 males were picked to found each generation, beginning with worms from the HC-founding stock. In parallel, for each of three independent NC lines, 60 L4 self-fertile hermaphrodites were picked to found each generation, beginning with worms from the NC-founding stock. Both HC and NC lines were allowed to evolve in this manner for 60 generations, with worms frozen for later analysis at generations 30 and 60. After experimental evolution was halted, worms from the evolved lines were thawed and examined for the following male phenotypes: sperm size, sperm competitive ability, male mating behavior, and effects of male exposure on hermaphrodite mortality rates.

### Life history assays

To determine population-specific effects of mating on female lifespan in *C. remanei*, the survivorship of age-synchronized cohorts of virgin and mated females was compared, with mated females receiving either a short-term (24 h) access to males, or with females having life-long access to males. For unmated/virgin longevity, egg-laying females from each strain were picked randomly from the stock plates to fresh 90-mm Petri dishes (30 females per plate, three plates per strain) for three hours to obtain age-synchronized offspring, and then removed. When the synchronous offspring had molted to L4 larvae, virgin females were identified and transferred to individual 30-mm dishes (65 EM464 females, 40 PB259 females, and 60 SB146 females, respectively). Individuals were transferred daily to fresh dishes and checked for mortality. A female was scored as dead when it showed no signs of movement, no pharyngeal pumping (a feeding behavior), and did not respond to light prodding with a fine platinum wire.

For the short term (24 h) mating assays, procedures were as above except that when individuals had molted to the L4 stage, they were identified to sex, and 5 males and one female were each placed on a 30-mm Petri dish. All possible combinations of the three strains were crossed, to examine possible interaction effects between males and females of different genetic backgrounds. All males were removed after 24-h, and the females then transferred daily during their egg-laying period, and less frequently in their post-reproductive period [37]. Daily mortality was scored as above. For the long term mating assays, males were maintained throughout the female lifetime. Extra males from the age-synchronized plates were supplied as needed to replace dead or injured males to maintain constant male density, and plates were inspected daily for female mortality.

For all of the crosses described above, female fecundity was calculated by reserving all plates containing eggs and counting the offspring under a dissecting microscope. To facilitate this and diminish counting errors, these plates were incubated at 20 °C until the offspring were at least L4 larvae in size. Because of this delay in counting, any inviable eggs or juvenile mortality were discounted in estimates of female fecundity. Mating effects were estimated using JMP 10 (SAS Institute) via a 3-way factorial ANOVA with female strain, male strain, and length of male access as fixed effects. Posthoc tests were conducted using Tukey’s Honest Significant Difference (HSD) procedure.

Male effects on hermaphrodite longevity within *C. elegans* were assessed using a mass-mating assay in which ten wildtype hermaphrodites were placed with 20 males from one of the following sources: an HC line, an NC line, CB4856 (Hawaiian isolate), N2 (Bristol, England isolate), the original mixed stock from which the HC lines were founded, or the original mixed stock from which the NC lines were founded. The investigator was always blind to the source of the males. Every 24 h for 5 days, all living worms were transferred to fresh plates, any dead or missing males were replenished from source plates, and daily hermaphrodite deaths were scored. Differences in longevity were assessed using an ANOVA, with comparisons to the ancestor made via Dunnett’s procedure.

### Sperm size

For at least two generations prior to sampling sperm, worms were kept on uncrowded plates to insure abundant food and avoid dauer formation. Virgin L4 males were isolated 20–24 h prior to dissection, and dissected according to a standardized protocol [51] with electrolytically sharpened tungsten needles (*C. elegans*) or insect pins (*C. remanei*) in freshly thawed aliquots of SM1 adjusted to pH 7.0 and supplemented with 10 mg/ml polyvinylpyrrolidone (Sigma PVP40). Spermatid images were captured using Nomarski optics at 600X *(C. elegans*) or 400X *(C. remanei*) magnification. Cross-sectional areas of round spermatids (*i.e.*, chosen prior to any shape changes associated with spermatid activation) were measured using NIH Image or Image Pro (Media Cybernetics). For *C. remanei*, an average of 60 spermatids from ten different males were measured from each of the three different strains to estimate among-strain differences. For the *C. elegans* experimental evolution lines, ten spermatids were measured from each of 20 males (*i.e.*, 200 spermatids total) at each of three different times points (generations 0, 30, and 60). Differences in sperm size were analyzed via an analysis of variance (ANOVA) using JMP 10 (SAS Institute). For *C. remanei*, strain was the sole main effect. For *C. elegans*, a nested ANOVA model including competition treatment, replicate line (nested within competition), and generation was fit, with competition and generation as fixed effects, and replicate line as a random effect. Specific evolutionary hypotheses were tested using contrast coefficients as a subset of the overall model.

### Sperm competition

In *C. elegans*, self-sterile hermaphrodites from stock CB4856 harboring [*fog-2*] introgessions were used as the “arena” for male-male competition. To create standard competitor males, the [*mIsll IV*] transgene from stock PD4792 was introgressed into the AB2 stock genetic background by 10 generations of backcrossing with selection to maintain the array; worms harboring the transgene express Green Fluorescent Protein (GFP) in the pharynx muscles, which served as a dominant marker to score paternity in progeny (*i.e.*, worms expressing GFP were sired by AB2 [GFP] males, whereas worms lacking GFP expression were sired by the males being tested for competitive ability).

During the two generations prior to each sperm competition assay, all of the relevant stocks were refreshed on new plates at low densities to make sure that worms were consistently well-fed and did not spend any time as dauer larvae. Three days before the sperm competition assay began, 30–50 eggs from each stock were transferred to empty, fresh plates so that they could develop at controlled densities. About 24 h prior to the beginning of the assay, worms at a standardized L4 stage were moved to fresh plates and kept at low densities, away from members of the opposite sex, in order to control mating opportunities prior to the sperm competition assay.

To test sperm offense ability, one CB4856 [*fog-2*] self-sterile hermaphrodite was exposed to 5 AB2 [GFP] males for 24 h, then transferred to a new plate with 5 HC or 5 NC males for another 24-h (Day 1 plate). The hermaphrodite was then kept on a plate alone for 24-h (Day 2 plate) and then moved again to a fresh plate for a final 24-h period (Day 3 plate). Any hermaphrodite dying before the end of the experiment was excluded from the data set. Once the progeny on the Day 1 – Day 3 plates had reached maturity, the ratio of GFP to non-GFP progeny was counted (a minimum of 30 random progeny were counted for each plate per day) with the investigator blind to the source of male parents. For sperm defense, the same experimental design was employed, only switching the HC or NC males to the first day and the AB2 [GFP] males to the second day.

In *C. remanei*, sperm competition assays were carried out essentially the same as above, except that *C. remanei* females were used as the “arena” for male-male competition, and males from the three strains were allowed to compete directly (*i.e.*, no standardized male competitors), with offspring paternity determined by examining a microsatellite marker that varied among strains. Specifically, a tetranucleotide microsatellite, (GACA)13, was identified from contig 52.84, indices 6754–6807, of the *C. remanei* Pcap Asembly v2 (Genome Sequencing Center, Washington University School of Medicine, St. Louis, Missouri, USA) using Tandem Repeats Finder v 3.21 [52]. Primers flanking this repeat, GGAACAGATGAGGTGATGACG and CATCTCCGCTCTCCAATGA, were designed in Primer Designer v 2.0 (Scientific and Educational Software, Cary, NC). EM464 and SB146 are fixed for different alleles at microsatellite locus cr52.84, approximately 210 bp and 180 bp respectively, allowing paternity assignment of F1 progeny resulting from crosses between these strains.

Sperm competitive ability was measured both as the ability of a focal male to displace the sperm of the first male when introduced as the second male (“offense” or P2; [53]) and to avoid displacement of sperm from the second male when introduced as the first male (“defense” or P1). These were not measures of single mating sperm displacement, since it was likely that the males and females mated multiple times during the day that they were together. “Day 1” of sperm competition was the first day when both types of competing sperm were present within the same female. On days 2–3, no males were present on the plates with females, so any offspring produced on these days were generated from residual sperm remaining from the initial matings. For *C. remanei*, male-specific effects were tested by comparing P1 and P2 measures in a particular cross combination in order to estimate whether males from a particular strain performed better as first and/or second males compared to those of another strain. This effect was tested using Fisher’s Exact Test of the sperm counts for each male. For *C. elegans*, sperm competitive ability relative to the fixed GFP-tester line was compared between the high-competition and no-competition lines using offspring counts within a nested logistic-regression that tests the effect of the experimental treatment while taking into account variance among experimental replicates (JMP 10, SAS Institute).

### *C. elegans* male mating behavior

One virgin male from either an HC or NC line was placed on an OP50 bacterial spot (~7 mm diameter, grown overnight) with ten virgin, four-day-old wildtype hermaphrodites from N2 Bristol, and observed for twenty minutes. The observer was blind to the source of the male. Successful matings within twenty minutes were noted, and for these matings the time until spicule insertion and the duration of spicule insertion were recorded.

### Availability of supporting data

The data sets supporting the results of this article are available in the Dryad Data Repository (www.datadryad.org), doi:10.5061/dryad.b13f4.
